# In Vitro Activity of Propolis on Oral Microorganisms and Biofilms

**DOI:** 10.3390/antibiotics10091045

**Published:** 2021-08-26

**Authors:** Alexandra Stähli, Hannah Schröter, Simonetta Bullitta, Francesca Serralutzu, Antonio Dore, Sandor Nietzsche, Egle Milia, Anton Sculean, Sigrun Eick

**Affiliations:** 1 Department of Periodontology, School of Dental Medicine, University of Bern, 3010 Bern, Switzerland; alexandra.staehli@zmk.unibe.ch (A.S.); hannah.schroeter@gmx.ch (H.S.); anton.sculean@zmk.unibe.ch (A.S.); 2 C.N.R., Institute for Animal Production System in Mediterranean Environment (ISPAAM), Traversa La Crucca 3, Località Baldinca, 07100 Sassari, Italy; simonettamaria.bullitta@cnr.it (S.B.); francesca.serralutzu@cnr.it (F.S.); 3 C.N.R., Institute of Sciences of Food Production (ISPA), Traversa La Crucca 3, Località Baldinca, 07100 Sassari, Italy; antonio.dore@cnr.it; 4 Center for Electron Microscopy, Jena University Hospital, 07743 Jena, Germany; Sandor.Nietzsche@med.uni-jena.de; 5 Department of Medicine, Surgery and Experimental Sciences, University of Sassari, 07100 Sassari, Italy; emilia@uniss.it

**Keywords:** propolis preparations, minimal inhibitory concentrations, oral multi-species biofilm, periodontal disease, caries, candidiasis

## Abstract

Natural products are being discussed as alternatives to commonly used chemicals in antimicrobial therapy. The study aimed to investigate the antimicrobial activity of propolis against microbial species associated with caries, periodontal disease, and Candida infections. Two commercially available ethanolic extracts of Brazilian and one of European propolis (EEP) were used. The minimal inhibitory concentrations (MIC) of propolis and controls against eight microbial strains were determined. Scanning and transmission electron microscopy (SEM and TEM) images visualized the effect of propolis on microorganisms. Subsequently, the activity on three different multi-species biofilms (both formation and existing biofilms) was assessed. All MIC values of the Brazilian EEPs were low against the tested oral species (≤0.1 mg/mL–3.13 mg/mL propolis (*Candida albicans*)). The European EEP had slightly higher MICs than the Brazilian EEPs. The SEM and TEM images suggest an interaction of propolis with the microbial cell wall. The European EEP exhibited the strongest effect on retarding biofilm formation, whereas the Brazilian EEPs were highly active against preformed biofilms (100 mg/mL propolis of both EEPs reduced colony forming unit counts always by more than 6 log10). The antimicrobial and anti-biofilm activities point to the potential of propolis as an adjunct in oral health care products.

## 1. Introduction

Microorganisms are an etiological factor in most common oral diseases, caries, and periodontal disease, as well as *Candida* infection. The pathogenesis of all these diseases is associated with a disturbed homoeostasis of the microbiota induced by environmental factors. In caries, for example, the presence of sucrose initiates a shift in the microbiota towards the dominance of acidogenic and aciduric bacteria (including mutans streptococci) which may lead to mineral loss of the teeth [1]. Periodontal diseases cover dental plaque biofilm-induced gingivitis, affecting only the gingiva [2], and periodontitis, which is characterized by dysbiotic plaque biofilms and by progressive destruction of the periodontium [3]. In the case of candidiasis, systemic diseases such as diabetes mellitus, intake of antibiotics, immunosuppression, or local factors such as wearing a prosthesis are often present and contribute to an overgrowth of *Candida* sp. [4]. The use of antibiotics in dentistry should be restricted due to the emerging increase in resistance [5], but also due to common adverse effects such as opportunistic yeast infections and gastrointestinal complications [6]. Among the antiseptics, the gold standard is still chlorhexidine digluconate. However, side effects such as burning sensations or altered taste frequently occur [7]. Meanwhile the cytotoxicity of the compound has been confirmed in several in vitro studies [8,9]. Moreover, chlorhexidine may contribute to cross resistance in antibiotics [5].

Thus, there is a need to search for alternatives in antimicrobial therapy. Natural products are being increasingly discussed as alternatives to the commonly used chemicals. Over the last years, they have been added to several oral health-care products. As such, propolis, a resinous exudate collected by honey bees and mixed with bee wax [10], has come into the focus of researchers and clinicians.

Propolis consists of about 50% resins and balsam, 30% bee wax, 5% pollen, 10% essential and aromatic oils, and some other substances [11]. Up to 300 different components have been identified in propolis; its biological activity is linked with polyphenols (flavonoids, phenolic compounds) and terpenoids [11]. Flavonoids present in propolis are chrysin, pinocembrin, apigenin, galangin, kaempherol, quercetin, tectochrysin, pinostrobin, and pinobenchin; phenolic acids found are ferulic acid, cinnamic acid, caffeic acid, benzoic acid, salicylic acid, *p*-cumaric acid, the esters artepillin C and caffeic acid phenethyl ester (CAPE), and representatives of terpenoids such as camphor, terpineol, geraniol, nerol, and farnesol [11].

It is discussed to provide health benefits in gastrointestinal disorders, bacterial vaginosis, vulvovaginal candidiasis, dermatological care (wound healing), oncologic treatment, and in oral diseases [12]. Recently, it has been proposed as a therapeutic option in the treatment of COVID-19 [13]. In dentistry, propolis has been studied in endodontic infections [14], for caries prevention [15], in periodontal therapy [16], and in denture stomatitis associated with *Candida* ssp. [17]. In vitro, antimicrobial and anti-biofilm activity by selected propolis were shown [18,19,20].

This study aimed to compare the antimicrobial activity of three ethanolic extracts of propolis (EEP) originating from South America (red and green Brazilian propolis) and from Central Europe on microbial species associated with caries, periodontal disease and with Candida infections. The minimal inhibitory concentrations (MIC) of propolis against these microorganisms were determined, and then activity of EEPs on three different multi-species biofilms was assessed. 

## 2. Results

In microbiological assays, the concentration values for propolis represent the respective mg/mL of propolis, and not of the EEPs. However, it has to be admitted that all propolis were dissolved in ethanol. Therefore, ethanol was always included as a control. In the biofilm assays, the used 35% of ethanol corresponded to 100 mg/mL propolis of the Brazilians EEPs and to 175 mg/mL propolis of the European EEP. Further, a chlorhexidine digluconate solution without additives (positive control) and a NaCl-solution (0.9% *w/v*; negative control) were included.

In the biofilm assays, both the effect of EEPs on biofilm formation (coating of the surface with the EEP or control), as well as on a preformed biofilm, were studied.

### 2.1. Antimicrobial Activity against Planktonic Microorganisms

All tested EEPs were highly active against the tested oral strains. The European EEP seemed to act slightly less inhibitory, the MICs against *Actinomyces naeslundii* and *Prevotella intermedia* were five and three dilution steps higher than those of the Brazilian EEPs, respectively. The MICs of ethanol most clearly exceeded the corresponding MICs of EEP, thus the antimicrobial activity can be attributed to propolis (Table 1).

### 2.2. SEM and TEM Images of Microorganisms Exposed to Propolis

Scanning electron microscopy (SEM) and transmission electron microscopy (TEM) images were prepared from selected microbial strains after having been exposed to 25 mg/mL European EEP and green Brazilian EEP.

**Figure 1:**
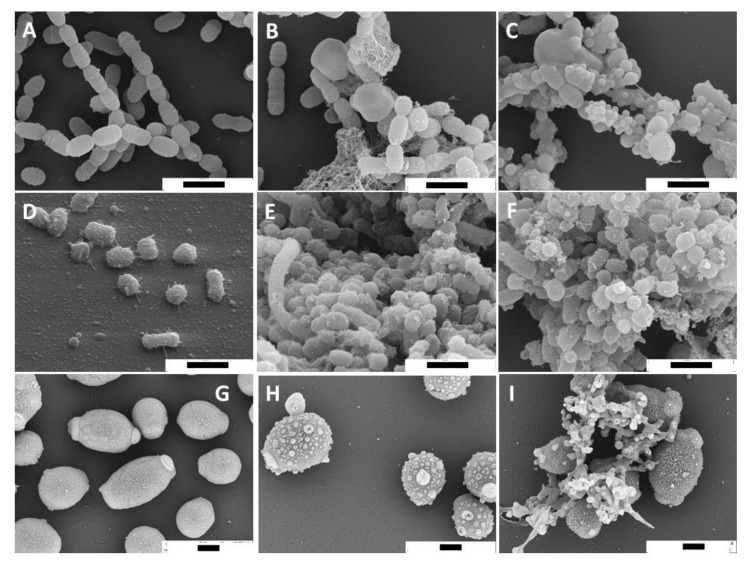
Scanning electron microscopy images of *Streptococcus mutans* (**A**–**C**), *Porphyromonas gingivalis* (**D**–**F**), and *Candida albicans* (**G**–**I**) without (**A**,**D**,**G**) and with 5 min exposure to 25 mg/mL propolis of the different ethanolic propolis extracts (European: (**B**,**E**,**H**); green Brazilian: (**C**,**F**,**I**)). Bar (**A**–**I**) 1 µm.

The SEM images suggest an interaction with the microbial cell wall. Small until large vesicles attached to the cell wall surface are visible after exposure to European EEP. In particular, green Brazilian EEP seemed to stick damaged cells together (Figure 1).

**Figure 2:**
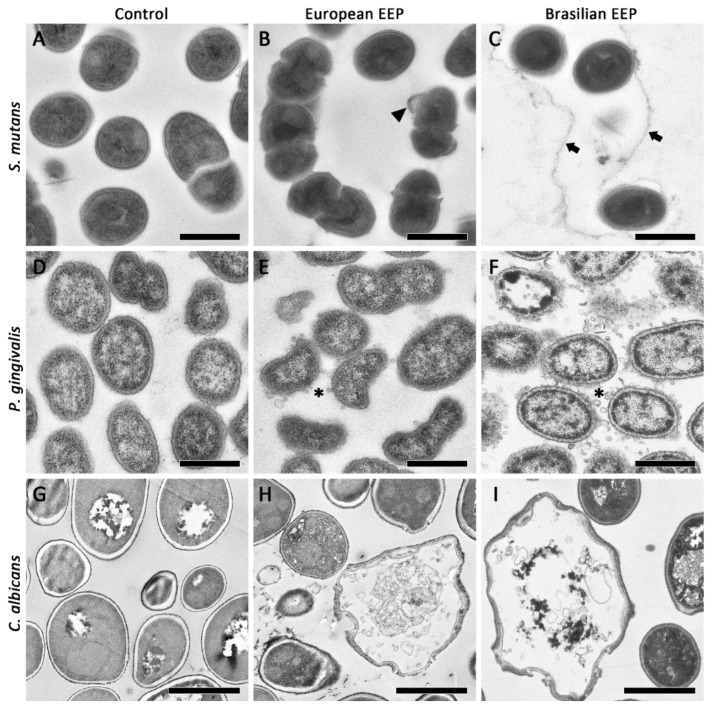
Transmission electron microscopy images of *Streptococcus mutans* (**A**–**C**), *Porphyromonas gingivalis* (**D**–**F**), and *Candida albicans* (**G**–**I**) without (**A**,**D**,**G**) and with 5 min exposure to 25 mg/mL propolis of the different ethanolic propolis extracts (European: (**B**,**E**,**H**); Brazilian green: (**C**,**F**,**I**)). Bar (**A**–**I**) 500 nm for bacteria, 2 µm for *C. albicans*; After exposure to propolis there are attachments visible (arrowhead), an enclosing compartment—maybe made of EPS (arrows), and a lot of small (40 nm) vesicles (asterisks).

The TEM images show a minor modification on *Streptococcus mutans* cells (Figure 2A–C). Rarely, bubbles attached to the cell surface are visible. In case of *Porphyromonas gingivalis*, vesicles outside of the cells appeared, and in parts only sheaths of bacteria are detected (Figure 2D–F). The TEM images of *Candida albicans* show enlarged cells and suggest a loss of the cell wall integrity after exposure to propolis (Figure 2G–I).

### 2.3. Cariogenic Biofilm

**Figure 3:**
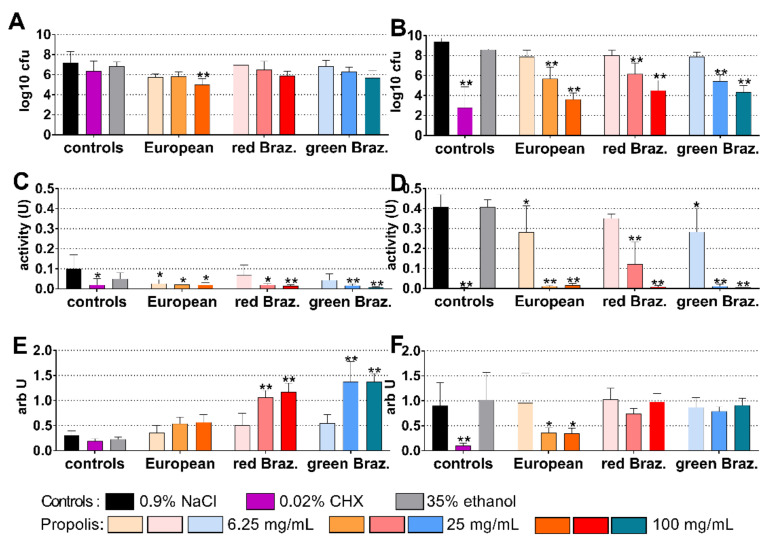
Activity of the different ethanolic extracts of propolis (European and red and green Brazilian (Braz.)) on colony forming units (cfu; (**A**,**B**)), metabolic activity (**C**,**D**) and quantity (**E**,**F**) in a formed cariogenic biofilm after 4 h (**A**,**C**,**E**) and 24 h (**B**,**D**,**F**). */** *p* < 0.05/*p* < 0.01 vs. control 0.9% NaCl.

The cariogenic control biofilm contained 7.21 log10 cfu after 4 h and 9.41 log10 after 24 h of biofilm formation. The European EEP was most inhibitory after 4 h: coating with 100 mg/mL propolis reduced bacterial counts by 2.21 log10 (*p* < 0.001 vs. control; Figure 3A). Among the EEPs, 100 mg/mL propolis of the European EEP were also most active after 24 h, the reduction was 5.78 log10 (*p* < 0.001 vs. control). However, it did not reach the inhibitory effect of 0.02% CHX; there the difference to control was −6.61 log10 (*p* < 0.001; Figure 3D).

The metabolic activity decreased after the application of 0.02% CHX (*p* = 0.010) and of all EEPs (except for 6.25 mg/mL propolis of both Brazilian EEPs) at 4 h (Figure 3B). At 24 h, the activity was clearly reduced by 25 mg/mL and 100 mg/mL of all propolis and by 0.02% CHX (each *p* < 0.001 vs. control; Figure 3E). At 4 h, the quantity of biofilm determined by crystal violet staining increased after both Brazilian EEPs, which might be related to a staining of the applied EEP (Figure 3C). At 24 h, however, there was a reduced quantity after 25 mg/mL (*p* = 0.029) and 100 mg/mL (*p* = 0.024) of the European EEP (Figure 3F). 

**Figure 4:**
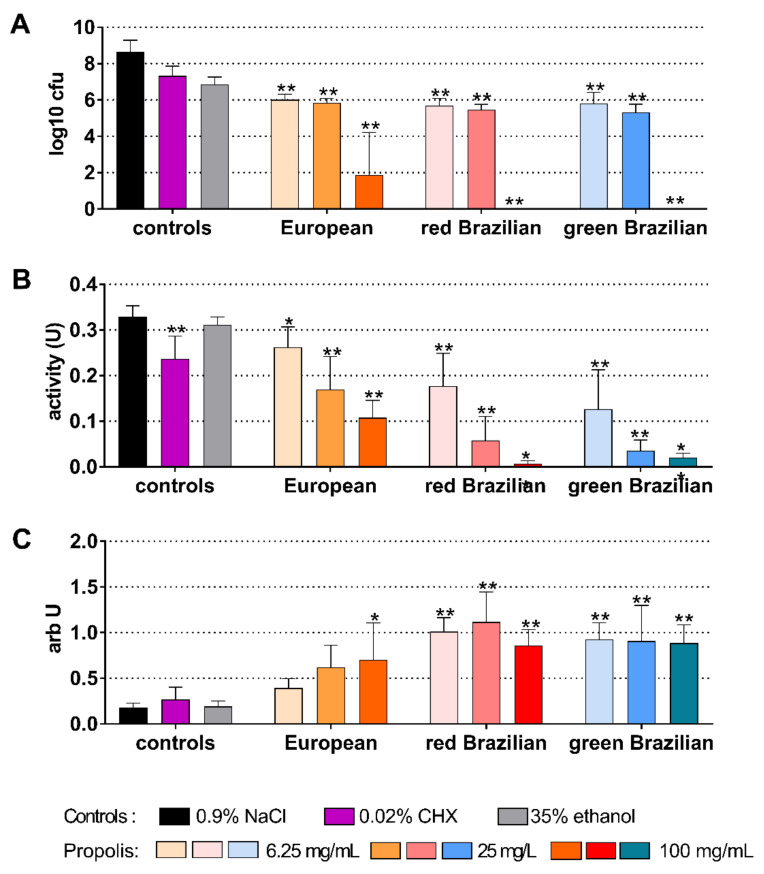
Activity of the different ethanolic extracts of propolis (European and red and green Brazilian) on colony forming units (cfu) (**A**), metabolic activity (**B**), and quantity (**C**) on a 48 h cariogenic biofilm. */** *p* < 0.05/*p* < 0.01 vs. control 0.9% NaCl.

When EEPs were tested on a 48 h biofilm, all EEPs were highly active and exhibited reduced concentration-dependent cfu counts (all concentrations vs. control each *p* < 0.001). After applying 100 mg/mL propolis of the Brazilian EEPs, no cfu were counted anymore (Figure 4A). The metabolic activity decreased after applying 0.02% CHX, and decreased concentration-dependently after all EEPs (6.25 mg/mL propolis of the European *p* = 0.005; all others *p* < 0.001 vs. control; Figure 4B). Higher quantities were measured after different EEP concentrations (Figure 4C).

### 2.4. Periodontal Biofilm

**Figure 5:**
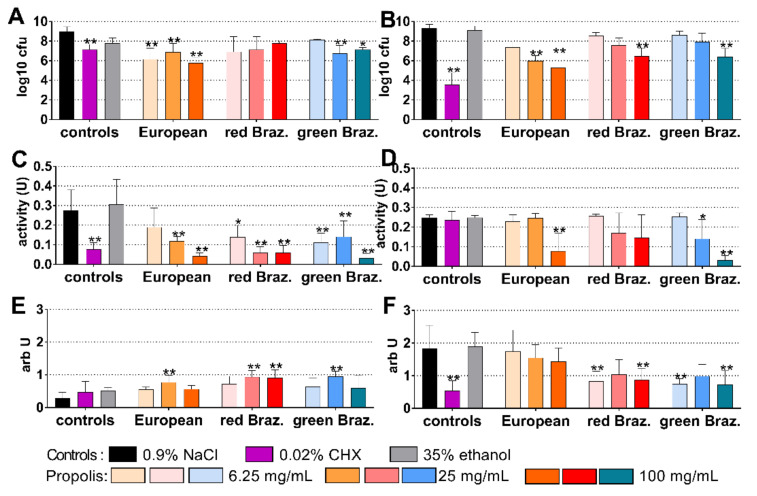
Activity of the different ethanolic extracts of propolis (European and red and green Brazilian (Braz.)) on colony forming unity (cfu) (**A**,**B**), metabolic activity (**C**,**D**), and quantity (**E**,**F**) in a formed periodontal biofilm after 4 h (**A**,**C**,**E**) and 24 h (**B**,**D**,**F**). */** *p* < 0.05/*p* < 0.01 vs. control 0.9% NaCl.

The periodontal biofilm contained 8.99 log10 cfu after 4 h and 9.34 log10 after 24 h of biofilm formation. The European EEP was most inhibitory after 4 h, and coating with 100 mg/mL propolis reduced bacterial counts by 3.21 log10 (*p* < 0.001 vs. control; Figure 5A). As before in cariogenic biofilm, CHX was most active after 24 h, followed by 100 mg/mL propolis of the European EEP, the respective reductions were 5.80 log10 and 4.03 log10 (each *p* = 0.001 vs. control; Figure 5D).

The metabolic activity decreased after the application of 0.02% CHX (*p* = 0.010) and of all EEPs except for 6.25 mg/mL propolis of the European EEP at 4 h (Figure 3B). At 24 h, the activity was reduced by 100 mg/mL propolis of the European EEP, as well as by 25 mg/mL (*p* = 0.025) and 100 mg/mL propolis (*p* < 0.001) of the green Brazilian EEP (Figure 5E). At 4 h, the quantity of biofilm increased in part after the addition of the EEPs (Figure 5C). At 24 h, there was a reduced quantity after 0.02% CHX (*p* < 0.001) and 6.25 mg/mL (*p* = 0.001; *p* = 0.003) and 100 mg/mL (*p* = 0.001; *p* = 0.006) propolis of the European EEP and green Brazilian EEP (respectively) (Figure 5F).

**Figure 6:**
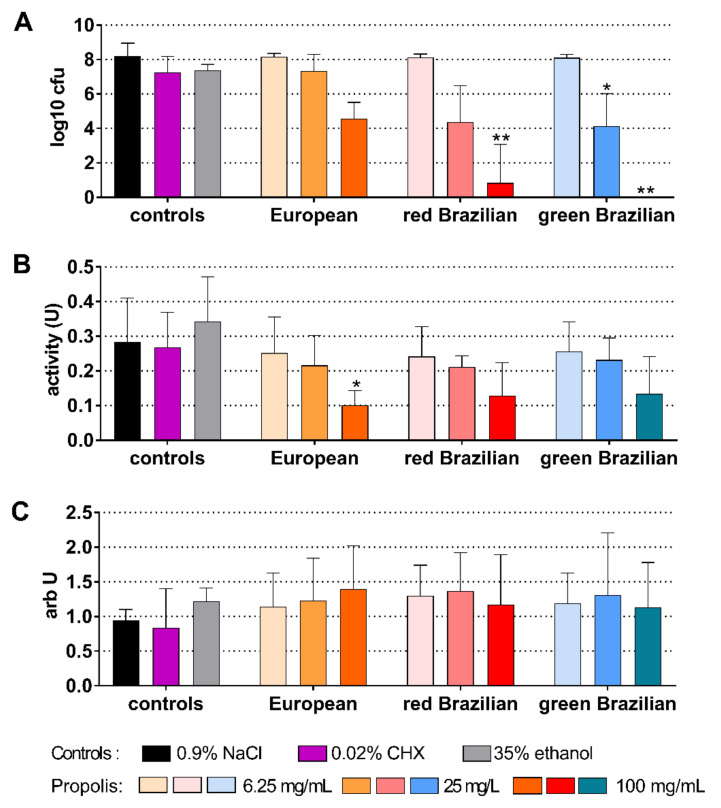
Activity of the different ethanolic extracts of propolis (European and red and green Brazilian) on colony forming units (cfu) (**A**), metabolic activity (**B**), and quantity (**C**) on a 5-day-old periodontal biofilm. */** *p* < 0.05/*p* < 0.01 vs. control 0.9% NaCl.

When the activity of EEP was tested on a 5-day-old biofilm, only higher concentrated Brazilian EEPs were able to reduce bacterial counts. One hundred milligram per millilitre propolis of the green Brazilian EEP eliminated all cfu, and 100 mg/mL propolis of the red Brazilian EEP reduced the cfu counts by 7.36 log10 (both *p* < 0.001; Figure 6A). The metabolic activity decreased only after applying 100 mg/mL propolis of the European EEP (*p* = 0.012; Figure 6B). The quantity of the biofilms did not change (Figure 6C).

### 2.5. Candida Biofilm

**Figure 7:**
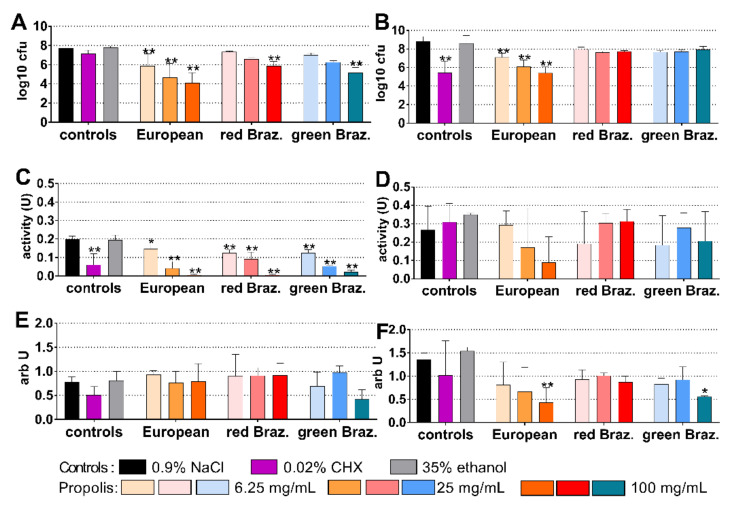
Activity of the different ethanolic extracts of propolis (European and red and green Brazilian (Braz.)) on colony forming units (cfu) (**A**,**B**), metabolic activity (**C**,**D**), and quantity (**E**,**F**) in a formed Candida biofilm after 4 h (**A**,**C**,**E**) and 24 h (**B**,**D**,**F**). */** *p* < 0.05/*p* < 0.01 vs. control 0.9% NaCl.

The formed Candida biofilm contained 7.74 log10 cfu after 4 h and 8.81 log10 cfu after 24 h. The European EEP was most inhibitory after 4 h, coating with 100 mg/mL reduced microbial counts by 3.65 log10 (*p* < 0.001 vs. control; Figure 7A). After 24 h, both 100 mg/mL propolis of the European EEP and 0.02% CHX were most active and reduced the cfu counts by about 3.4 log10 (each *p* < 0.001 vs. control; Figure 7D).

The metabolic activity decreased after the application of 0.02% CHX (*p* < 0.001), and of all EEPs (6.25 mg/mL propolis of the European EEP: *p* = 0.023, all others *p* < 0.001 vs. control each) (Figure 7B). At 24 h, there was no difference to the untreated control (Figure 7E). At 4 h, the quantity of the biofilm was unchanged (Figure 7C). At 24 h, there was a reduced quantity after 100 mg/mL propolis of EEP European (*p* = 0.001) and of the green Brazilian EEP (*p* = 0.011) (Figure 7F).

**Figure 8:**
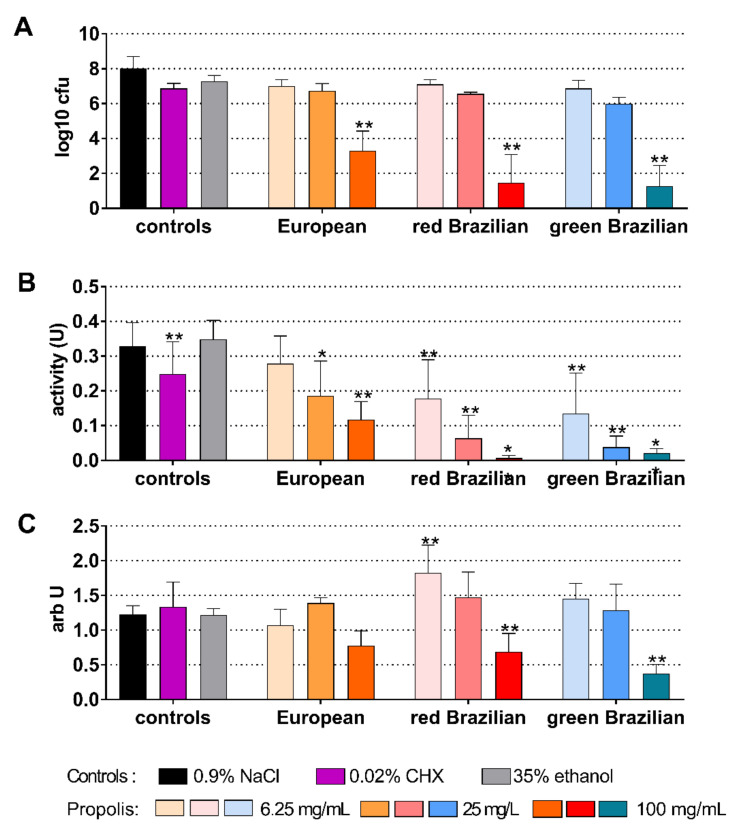
Activity of the different ethanolic extracts of propolis (European, Brazilian red, and Brazilian green) on colony forming units (cfu) (**A**), metabolic activity (**B**), and quantity (**C**) on a 5-day old Candida biofilm. */** *p* < 0.05/*p* < 0.01 vs. control 0.9% NaCl.

When the activity of the EEPs was tested on a 5-day-old biofilm, the 100 mg/mL propolis of the EEPs were able to statistically significantly reduce (all *p* < 0.001) the cfu counts. The reductions were between 4.72 log10 (European EEP) and 6.02 log10 (green Brazilian EEP; Figure 8A). The metabolic activity decreased after the application of 0.02% CHX (*p* = 0.009) and after all EEPs (each *p* < 0.001 vs. control) except for 6.25 mg/mL propolis of European EEP (Figure 8B). Biofilm quantity increased after 6.25 mg/mL propolis of the red Brazilian EEP (*p* = 0.001). Lower biofilm quantity was measured after 100 mg/mL propolis of the red Brazilian EEP (*p* = 0.005) and the green Brazilian EEP (*p* < 0.001) (Figure 8C).

### 2.6. Chemical Analysis of Propolis Extracts

The propolis extracts were subjected to a chemical analysis using a liquid chromatography—high resolution mass spectrometry (LC-HRMS).

The most relevant peaks were identified for each propolis sample and are presented in Table 2 with the corresponding retention times. Among the 22 compounds identified in the European propolis, 15 were flavonoids and 7 were phenolic acids and their esters. In the two Brazilian propolis samples, 10 phenolic acids, 12 flavonoids, and 2 prenylated derivatives of *p*-coumaric acid and acetophenone were found. The two Brazilian propolis showed the same composition, while only five compounds, luteolin 5-methyl ether, chrysin, benzyl caffeate, quercetin 5,3′-dimethyl ether, and caffeic acid phenethyl ester (CAPE), were also retrieved in the European propolis.

## 3. Discussion

In summary, EEPs were also active against oral microorganisms when organized in a biofilm. The European EEP seemed to be most inhibitory against biofilm formation, whereas the Brazilian EEPs might better combat an already formed biofilm. The EEPs inhibited the growth of oral microorganisms even in low concentrations.

Three different EEPs were included. The source of European propolis is mainly poplar, whereas the red Brazilian propolis originates from *Dalbergia ecastophyllum*, and the green Brazilian propolis is produced of resins from *Baccharis dracunculifolia* [21]. In the present study, the chemical analysis shows an equal profile for both Brazilian propolis, whereas there was a difference to the European propolis. A comparison of propolis from different regions found a higher flavonoid content in the European than in Brazilian propolis [22]. The analysis of the included three EEPs seems to confirm this, the European EEP was rich in quercetin, pinobanksin compounds, apigenin, and galangin. However, also regional variations exist between the European propolis. In northern Italy, for example, propolis from hilly regions is richer in *p*-cumaric acid and ferulic acid than those from plains [23]. Besides geographical, there are also seasonal differences regarding the propolis composition. In red Brazilian propolis, there is a higher content of the flavonoids vitexin, luteolin, and apigenin, and a lower content of caffeic acid and quercetin when collected during the rainy season in comparison with the dry season [24]. In our study, commercially available EEPs were used, and neither information about the exact geographical region, nor about the seasonal timepoint of collection was available. 

A few compounds have been identified in all three investigated EEPs. Among them is CAPE, whose antibacterial, anti-oxidative, and anti-inflammatory capacity has been extensively documented [25]. Here, we want to stress its activity against biofilm-forming oral cariogenic bacteria in general, and in particular its ability to reduce the metabolic activity of *S. mutans* in mature biofilms [26]. Besides CAPE, the antimicrobial activity found in the present study can also be attributed to the presence of quercetin and pinocembrin in both the Brazilian propolis, and to apigenin in the European propolis. In fact, CAPE, together with pinocembrin, apigenin, and quercetin, even at low concentrations, have previously been shown to exert antimicrobial activity against biofilm [27]. Additionally, Ristivojević et al. [28] pointed out that phenolic compounds such as pinocembrin, pinobanksin, chrysin, and galangin, as well as caffeic and ferulic acid esters (all present in our propolis samples), show a significant contribution to the antimicrobial activity of poplar-type propolis against Streptococcus species and Candida isolates (*C. albicans*, *C. glabrata*, and *C. krusei*). However, pinocembrin and apigenin in Chilean propolis demonstrated greater activity in comparison to the other compounds. The same compounds have also demonstrated extensive capacity against periodontal bacteria [29]. High content of flavonoids also exerted high anti-staphylococcal activity [30]. Several flavonoids were found in the propolis studied here.

All the MIC values were low against the tested oral species. The geographical origin also affected the antimicrobial activity of propolis. The European EEP seemed to be slightly less active than the Brazilian EEPs. This is in contrast to a recent study, where European propolis was found to have a higher antimicrobial activity against oral anaerobes than a Brazilian propolis [22]. In the present study, there was no difference between Gram-positive and Gram-negative bacteria; only *C. albicans* was negligibly less susceptible. In general, oral bacteria are highly susceptible to EEP [31,32]. The obtained results confirm a previous report on green Brazilian propolis with a strong growth inhibition of *S. mutans*, *Streptococcus sanguinis*, and *P. gingivalis* [19]. Our results are similar to a recent report on red propolis, where antibacterial activity against oral streptococci and lactobacilli was tested [20]. In the present study, the EEPs were highly active against *C. albicans* ATCC 76615 (MICs below 10 mg/mL of EEP each). However, a study which tested 50 propolis from Northern Poland against five *C. albicans* strains found in one fifth MICs of >25 mg/mL [33]. 

The SEM and TEM images suggest the microbial cell wall as the target of propolis. In case of *P. gingivalis* the mode of action was described as a membrane-rupturing effect [34]. Our TEM images may confirm this; in particular, after adding green Brazilian EEP, many vesicles closely located to the outer membrane occurred.

In the present study, three different multi-species biofilm models, a “cariogenic” biofilm, a “periodontal”, and a “*Candida*” biofilm were included. The effect on both biofilm formation (application on a surface free of microorganisms) and on an already existing biofilm was studied. In the experiments on biofilm formation, surfaces were coated with the EEPs and after the addition of culture media there were final concentrations of 0.0625% to 1% EEP in the assays. The present in vitro experiments showed that the European EEP exhibited the most retarded biofilm formation. The effect might be related to the high concentration of ferulic acid. Ferulic acid was shown to inhibit biofilm formation of *Pseudomonas aeruginosa* together with the inhibition of extracellular polysaccharides synthesis [35]. After 4 h of biofilm formation, the total microbial counts were lower when compared to CHX in all three biofilm models. At 24 h, CHX seemed to be more active than EEP. CHX is well known for its long lasting substantivity on surfaces [36]. EEP prevents adhesion of bacteria to surfaces. Mostly, this was associated with a strong reduction in the metabolic activity of the biofilm. The quantity of the biofilms, however, was less affected. After 4 h, often higher values of EEPs vs. controls were measured and, after 24 h of biofilm formation, there was only a reduction in part. This might be explained by a possible attachment of the applied EEP to the biofilm matrix. In the case of an already formed biofilm, the Brazilian EEPs were more active. It can be speculated that these EEPs affect components of the biofilm matrix, as Brazilian EEPs are rich in chlorogenic acid, which was shown to disrupt biofilm formation of *Klebsiella pneumoniae* [37].

It is noteworthy, that the “cariogenic” biofilm was more affected by the EEPs than the periodontal and the *Candida* biofilms. *S. mutans* and *Streptococcus sobrinus* were among the selected bacteria for the cariogenic biofilm; both synthesize water-insoluble extracellular polysaccharides. EEP was shown to decrease the adherence of *S. mutans* and *S. sobrinus* to surfaces; it inhibited water-insoluble glucan formation by targeting glucosyl transferase already at concentrations of 50 µg/mL [32]. Kouidhi et al. [31] tested the effect of propolis on adhesion of many oral streptococci and on a preformed biofilm. As in our study, they found a strong inhibition of bacterial adhesion, but also an activity on an already formed single-species biofilm. In the present study, an activity of EEP already in low concentrations was seen on the preformed cariogenic biofilm. The results are similar to a recent study on red Brazilian propolis, where the activity of 0.8% propolis was similar to those of 0.12% CHX; it is of interest to note that the addition of 500 ppm sodium fluoride did not affect the antibiofilm activity of that EEP [20]. Different results were found on a six-species biofilm consisting, among others, of *S. mutans* and *C. albicans*; a mouth rinse containing 10% propolis did not have any effect, whereas a reduction was seen after applying a toothpaste with 0.9% propolis [38].

Periodontal biofilm formation was inhibited by the EEPs but the effect on the preformed biofilm was very limited. The microbial counts of the biofilm (higher than those of the cariogenic biofilm) suggest a more complex biofilm. No bacterium able to form branched extracellular polysaccharides was used. Therefore, the results point towards glucans as the target of the EEP in cariogenic biofilms. A multispecies periodontal biofilm consisting of about 30 species was used by Miranda et al. [39], who showed that the total counts decreased by about 1 log10 and the metabolic activity by about 50% after applying 0.16% red Brazilian propolis.

The *Candida* biofilm consisting of two oral bacteria and *C. albicans* was also more susceptible to EEP than the periodontal biofilm. In biofilms formed by *C. albicans*, besides proteins, lipids, and extracellular DNA, carbohydrates contribute to 25% of the biofilm matrix. Of the latter a mannan–glucan complex was most abundant [40].

This study used different biofilm models. The good anti-biofilm activity may support the results of the few clinical reports. One gel with an unknown percent of propolis reduced marginal plaque to a higher extent compared with a regular toothpaste and, moreover, it also inhibited reformation of plaque [41]. A mouth rinse containing 2% of propolis applied in experimental gingivitis did not show a difference to the used positive control of 0.05% cetylpyridinium chloride [42]. In comparison with chlorhexidine digluconate, propolis was equally efficient in the treatment of gingivitis and increased the salivary anti-oxidant capacity [43]. A toothpaste containing 3% of the green Brazilian propolis was beneficial in improving plaque and gingival indices in patients undergoing orthodontic treatment [44] or having implant-supported prosthodontic reconstructions [45]. An irrigation with a 30% EEP after scaling and root planning reduced sites with bleeding on probing, but also bacteria incl. *P. gingivalis* [46]. Further, it is of interest to note that after a six-month oral intake of propolis capsules, not only did periodontal indices improve in type 2 diabetes patients, but the HbA1c level decreased by nearly 1% [47]. In denture stomatitis, a propolis gel was clinically equally effective as a miconazole gel, however a clear reduction in *C. albicans* counts was mostly found in the miconazole than in in the propolis group [17].

The advantages of our study are the inclusion of several oral microorganisms and the use of three different biofilm models. In the biofilm models, the focus was both on retarding biofilm formation and the activity on a preformed biofilm. It has to be mentioned, however, that the multi-species biofilm models do not reflect the complexity of biofilms in the oral cavity. Further, these models do not consider the interaction with host cells including immune cells. It is relevant to mention that some of the compounds (e.g., artepillin) present in the analyzed EEP possess antioxidant and anti-inflammatory properties [48]. Polish propolis, rich in pinocembrin, chrysin, galangin, and coumaric acid, was shown to exert not only antifungal, but also anti-oxidative and cytoprotective activities [49]. More research is needed to acquire better knowledge about the anti-inflammatory and anti-oxidative effects of propolis related to oral diseases.

Our in vitro results underline the potential of propolis as an adjunct in oral health care products. The experiments focused on the antimicrobial including antibiofilm aspect. All of the three commercially available EEPs used were active, and thus the question of which propolis or which combination might be the best to be included in an oral health care product cannot be answered now, and should be a focus in further studies.

## 4. Materials and Methods

### 4.1. Propolis Preparations

EEPs originating from South America (Extrato de Própolis (red Brazilian), Extrato de Própolis verde (green Brazilian), both APIS Flora, Sāo Paulo, Brasil) and Central Europe (Apinatura Propolistinktur (European), APINATURA, Naters, Switzerland) were used. The Brazilian EEPs were 30% propolis in ethanolic preparation, the European EEP contained 50% propolis. A chlorhexidine digluconate solution without additives was used as a positive control and a 35% ethanolic solution (equivalent 10% Brazilian propolis) and NaCl-solution (0.9% *w/v*) as negative controls.

### 4.2. Microorganisms

The following microorganisms were included in the experiments:

1. *Streptococcus gordonii* ATCC 10558
2. *Actinomyces naeslundii* ATCC 12104
3. *S. mutans* ATCC 25175
4. *S. sobrinus* ATCC 33478
5. *Lactobacillus acidophilus* ATCC 11975
6. *Fusobacterium nucleatum* ATCC 25586
7. *Campylobacter rectus* ATCC 33238
8. *Parvimonas micra* ATCC 33270
9. *Eikenella corrodens* ATCC 23834
10. *Prevotella intermedia* ATCC 2561
11. *Capnocytophaga gingivalis* ATCC 33624
12. *Porphyromonas gingivalis* ATCC 33277
13. *Tannerella forsythia* ATCC 43037
14. *Filifactor alocis* ATCC 33099
15. *Treponema denticola* ATCC 35405
16. *Candida albicans* ATCC 76615.

Eight strains were included in the MIC determinations. All strains were used in biofilm assays (5 in the cariogenic biofilm, 12 in the periodontal biofilm, and 3 in the *Candida* biofilm).

### 4.3. Susceptibility Tests: Determination of MICs

Micro-broth dilution technique was used to determine minimal inhibitory concentration [50] values against *S. gordonii* ATCC 10558, *A. naeslundii* ATCC 12104, *S. mutans* ATCC 25175, *F. nucleatum* ATCC 25586, *P. micra* ATCC 33270, *P. intermedia* ATCC 2561, *P. gingivalis* ATCC 33277, and *C. albicans* ATCC 76615. After subcultivation of microbial strains and checking of purity, a defined inoculum was added to the broth containing defined concentrations of the EEP formulation as a two-fold dilution series or the controls. After an incubation time of 42 h (18 h aerobes), the growth of microbes was analyzed by visual checking of turbidity (and, if necessary, by subcultivation). MIC represented the lowest concentration without visible turbidity.

These experiments were made in independent replicates.

### 4.4. Visualization of Mode of Action of Propolis

*S. mutans, P. gingivalis*, and *C. albicans* suspensions were prepared. Then, they were exposed for 5 min to European EEP or green Brazilian EEP, which corresponds to a concentration of 25 mg/mL propolis or 0.9% w/v NaCl. Thereafter, the samples underwent transmission electron microscopy [51] and scanning electron microscopy (SEM).

The samples were centrifuged and washed twice with 0.9% *w*/*v* NaCl and finally suspended to a 20-fold concentration of microorganisms. For SEM, this suspension was transferred to slides and thereafter fixed with 2% glutaraldehyde solution in 0.1 M cacodylate buffer (pH 7.4) for 15 min. Then, samples were washed 3-fold with 0.1 M cacodylate buffer and dehydrated in ascending ethanol concentrations (30, 50, 70, 90, and 100%) for 10 min each. Subsequently, the samples were critical-point dried using liquid CO_2_ and sputter coated with platinum (thickness approx. 1 nm) using a SCD005 sputter coater (BAL-TEC, Liechtenstein) to avoid surface charging. Finally, the specimens were investigated with a field emission (FE) scanning electron microscope LEO-1530 (Carl Zeiss NTS GmbH, Oberkochen, Germany).

For TEM, microbial suspensions were fixed with 0.5% formaldehyde and 2% glutaraldehyde in 0.1 M cacodylate buffer (pH 7.4) for 30 min. After washing 3-fold with 0.1 M cacodylate buffer and post-fixating with osmiumtetroxide for 1 h, dehydration in ascending ethanol series with post-staining in uranylacetate was performed. Afterwards, the samples were embedded in epoxy resin (Araldite) and sectioned using a Leica Ultracut E (Leica, Wetzlar, Germany). Ultrathin sections (60 nm thickness) were mounted on filmed Cu grids, post-stained with lead citrate, and studied in a transmission electron microscope (EM 900, Zeiss, Oberkochen, Germany) at 80 kV and magnifications of 3,000× to 20,000×.

### 4.5. Activity on Biofilms

Three different biofilms were created. The cariogenic biofilm consisted of *S. gordonii* ATCC 10558, *A. naeslundii* ATCC 12104, *S. mutans* ATCC 25175, *S. sobrinus* ATCC 33478, and *L. acidophilus* ATCC 11975. The periodontal biofilm was formed of *S. gordonii* ATCC 10558, *A. naeslundii* ATCC 12104, *F. nucleatum* ATCC 25586, *Campylobacter rectus* ATCC 33238, *P. micra* ATCC 33270, *E. corrodens* ATCC 23834, *P. intermedia* ATCC 2561, *C. gingivalis* ATCC 33624, *P. gingivalis* ATCC 33277, *T. forsythia* ATCC 43037, *F. alocis* ATCC 33099, and *T. denticola* ATCC 35405. The *Candida* biofilm was created with *S. gordonii* ATCC 10558 and *C. albicans* ATCC 76615.

Two different scenarios were tested, (a) application of an oral health care product after mechanical removal of biofilm (influence on biofilm formation) and (b) if there is any effect on a biofilm which has been already formed (established biofilm).

(a) Influence on biofilm formation: Wells of a 96-well plate were covered with 25 µL of test substances for 1 min. Then, 25 µL of protein solution (1.5% bovine serum albumin/0.67% mucin in phosphate buffered saline (PBS)) and thereafter microbial suspension mixed with nutrient broth (Wilkins–Chalgren broth, Oxoid, Basingstoke, UK) in a ratio (volume 1:9) was added. Here, the analysis was made after 4 h and 24 h of development of biofilm at 37 °C with 5% CO_2_ or in an anaerobic atmosphere (periodontal biofilm).

(b) Established biofilm: Wells were coated with 25 µL of protein solution (1.5% bovine serum albumin/0.67% mucin in PBS) per well, and thereafter with 250 µL of microbial suspension mixed with nutrient broth in a ratio (volume 1:9). The well-plates were incubated in the respective atmosphere for 48 h (caries biofilm) or 5 days (periodontal biofilm, *Candida* biofilm). In the case of the periodontal biofilm, *P. gingivalis* ATCC 33277, *T. forsythia* ATCC 43037, and *T. denticola* ATCC 35405 were added again after 3.5 d. Then, the already formed biofilms were treated with the 25 µL of the test substances for 1 min after removing nutrient broth and short washing. After 1 min, nutrient broth (225 µL) was added and the biofilms were analyzed after 1 h of incubation.

Analysis of biofilms: Biofilms were removed from the surface by scraping and ultrasonication. After mixing by pipetting, a serial dilution was made and the total cfu counts were assessed. In addition, quantification of the biofilms was made after staining with crystal violet according to recently published protocols [52] and the metabolic activity of the biofilm suspension was assessed using Alamar blue as a redox indicator [53].

All biofilm experiments were made in two independent experiments in each independent quadruplicates.

### 4.6. Statistical Analysis

The data were presented as mean and standard deviation [54]. In case of microbial counts log10 values were reported. Data were compared using a one-way analysis of variance (ANOVA) with post hoc comparisons of groups using Bonferroni corrections. Differences to the untreated control are presented. A *p*-value of 0.05 was considered to be statistically significant. SPSS 26.0 (IBM, Chicago, IL, USA) software was used for statistical analysis.

### 4.7. Chemical Analysis of Propolis by LC–HRMS

Chemical analyses of propolis were carried out with a high performance liquid chromatograph (Agilent 1100 LC System, Agilent Technologies, Santa Clara, CA, USA) equipped with a 150 mm × 2.1 mm × 5 µm particle size C18 column (Phenomenex Gemini C18) and a diode array detector (G1316A, Agilent) operating at 270 or 320 nm. The flow rate was 0.4 mL min^−1^ during a 60 min period with an injection volume of 10 µL. A linear gradient elution of solvent acetic acid 0.2% (A) and acetonitrile (B) was applied with the following program: 0 min, 10% B; 0–20 min, 10–20% B; 20–40 min, 20–40% B; 40–50 min, and 40–70% B. The column was equilibrated for 8 min prior to each analysis. Mass spectra were recorded with an OrbiTrap High Resolution Mass Spectrometer (Q Exactive) equipped with heated electrospray ionization probe HESI-II; (ThermoFisher Scientific, Bremen, Germany) operating in both positive and negative ion mode. Parameters of the ion source were as follows: spray voltage, 3.5 kV (POS) 3.9 kV (Neg); sheath gas flow rate 45 (arbitrary units); auxiliary gas, 10 (arbitrary units); sweep gas, 3 (arbitrary units); and capillary temperature at 320 °C. Full MS acquisition was performed with resolution power 70,000 FWHM with mass accuracy of 5 ppm. The MS parameters were: AGC target 1e^6^, maximum injection time (IT) 200 ms, and scan range 100–1500 m/z. The Xcalibur™ 3.1.66 software (Thermo Scientific, Bremen, Germany) was used to control the instruments and to process the data. Mass deviation were calculated as parts per million (calculated mass-experimental mass/calculated mass) and were found to be below 5 ppm.

Peaks were made by comparing the retention time of standard with those of the samples, on the basis of their UV-vis spectra high resolution mass spectra, phytochemicals library, and reference literature.

## 5. Conclusions

The present study has shown that commercially available EEPs of propolis are active against oral microorganisms associated with dental caries, periodontal diseases, and *Candida* infections. Their main target appears to be the microbial cell wall. 

The in vitro experiments suggest a retardation of biofilm formation but also, when used in high concentrations, a dissolution of an existing biofilm. The antimicrobial and anti-biofilm activities of the EEPs support their incorporation in oral health care products. 

## Figures and Tables

**Table 1:** MIC values of propolis (mg/mL) in the different ethanolic preparations (EEP), chlorhexidine digluconate (CHX; %), and ethanol (%).

Strain	CHX	Ethanol	European EEP	Red Brazilian EEP	Green Brazilian EEP
*Streptococcus gordonii* ATCC 10558	≤0.0004	17.5	0.2	≤0.1	≤0.1
*Actinomyces naeslundii* ATCC 12104	0.0016	8.75	50	≤0.1	≤0.1
*S. mutans* ATCC 25175	0.0016	17.5	0.2	3.13	0.2
*Fusobacterium nucleatum* ATCC 25586	0.0008	17.5	3.13	0.2	0.2
*Prevotella intermedia* ATCC 25611	≤0.0004	4.38	12.5	≤0.1	≤0.1
*Porphyromonas gingivalis* ATCC 33277	0.0016	35.0	0.2	0.2	0.2
*Parvimonas micra* ATCC 33270	0.0031	35.0	≤0.1	≤0.1	≤0.1
*Candida albicans* ATCC 76615	≤0.0004	17.5	6.25	3.13	3.13

**Table 2:** Chemical analyses of the ethanolic extracts of propolis.

European Propolis	Peak	RT (min)	Compound	Brazilian Propolis	Peak	RT (min)	Compound
P1	1	28.01	Ferulic acid	P2, P3	1	14.02	Chlorogenic acid
P1	2	29.16	3-Hydroxy-4-methoxycinnamic acid	P2, P3	2	21.9	4-Hydroxycinnamic acid
P1	3	36.06	3,4-Dimethoxycinnamic acid	P2, P3	3	27.28	Luteolin 7-rutinoside
P1	4	36.59	DMCA isomer	P2, P3	4	29.45	Baccharin
P1	5	38.57	Pinobanksin-methyl ether isomer	P2, P3	5	30.18	ND
P1	6	40.79	Pinobanksin 5-methyl ether	P2, P3	6	31.98	Aromadendrin 4’-methyl ether 7-rhamnoside
P1	7	44.12	Quercetin 3-methyl ether	P2, P3	7	34.85	3,4-Dicaffeoylquinic acid
P1	8	45.51	Chrysin 5-methyl ether	P2, P3	8	35.89	3,5-Dicaffeoylquinic acid
P1	9	45.93	Apigenin	P2, P3	9	37.36	1,5-Dicaffeoylquinic acid
P1	10	46.09	Kaempferol	P2, P3	10	38.61	Kaempferol 7-methyl ether 4’-glucoside
P1	11	46.55	Isorhamnetin	P2, P3	11	43.95	Di-Caffeoyl quinic acid isomer
P1	12	46.97	Luteolin 5-methyl ether	P2, P3	12	45.04	Quercetin
P1	13	47.34	Quercetin 5,3’-dimethyl ether	P2, P3	13	47.54	Luteolin 5-methyl ether
P1	14	47.85	Galangin-5-methylether	P2, P3	14	47.96	Pinocembrin
P1	15	48.91	Quercetin 3,7-dimethyl ether	P2, P3	15	48.49	Drupanin
P1	16	49.19	Prenyl caffeate	P2, P3	16	49.08	Viscidone
P1	17	49.73	Chrysin	P2, P3	17	49.67	Chrysin
P1	18	50.17	Benzyl caffeate	P2, P3	18	49.87	Pinocembrin-5-methyl ether
P1	19	50.3	Pinobanksin 3-butyrate	P2, P3	19	50.17	Benzyl caffeate
P1	20	50.69	Caffeic acid phenethyl ester CAPE	P2, P3	20	50.33	Kaempferol-7-methyl ether
P1	1	28.01	Ferulic acid	P2, P3	1	14.02	Chlorogenic acid
P1	2	29.16	3-Hydroxy-4-methoxycinnamic acid	P2, P3	2	21.9	4-Hydroxycinnamic acid
P1	3	36.06	3,4-Dimethoxycinnamic acid	P2, P3	3	27.28	Luteolin 7-rutinoside
P1	4	36.59	DMCA isomer	P2, P3	4	29.45	Baccharin
P1	5	38.57	Pinobanksin-methyl ether isomer	P2, P3	5	30.18	ND

(P2 red propolis, P3 green propolis).

## Data Availability

Data are available on request by the authors.
